# Do the digestive tract symptoms in eating disorder patients represent functional gastrointestinal disorders?

**DOI:** 10.1186/1471-230X-13-38

**Published:** 2013-02-28

**Authors:** Suzanne Abraham, John E Kellow

**Affiliations:** 1 Department of Obstetrics and Gynaecology and the Northside Clinic, Sydney Medical School, Royal North Shore Hospital, Sydney, Australia; 2 Departments of Gastroenterology and Medicine, Royal North Shore Hospital, University of Sydney, Sydney, Australia; 3 Department of Obstetrics and Gynaecology, University of Sydney, Royal North Shore Hospital, St Leonards, Sydney, NSW, 2065, Australia

**Keywords:** Eating disorders, Functional gastrointestinal disorders, Pelvic floor symptoms, Pelvic floor dyssynergia

## Abstract

**Background:**

Gastrointestinal (GI) symptoms are common in patients with eating disorders. The aim of this study was to determine, using factor analysis, whether these GI symptom factors (clusters) in eating disorder patients hold true to the Rome II classification of functional gastrointestinal disorders (FGIDs).

**Methods:**

Inpatients in a specialised eating disorder unit completed the Rome II questionnaire. Data from 185 patients were analysed using factor analysis of 17 questions cited as present in 30% to 70% of the patients.

**Results:**

Five factors emerged accounting for 68% of the variance and these were termed: ‘oesophageal discomfort’, ‘bowel dysfunction’, ‘abdominal discomfort’, ‘pelvic floor dysfunction’, and ‘self-induced vomiting’. These factors are significantly related to the Rome II FGID categories of functional oesophageal, bowel and anorectal disorders, and to the specific FGIDs of IBS, functional abdominal bloating, functional constipation and pelvic floor dyssynergia. Both heartburn and chest pain were included in the oesophageal discomfort factor. The ‘pelvic floor dysfunction’ factor was distinct from functional constipation.

**Conclusions:**

The GI symptoms common in eating disorder patients very likely represent the same FGIDs that occur in non-ED patients. Symptoms of pelvic floor dysfunction in the absence of functional constipation, however, are prominent in eating disorder patients. Further investigation of the items comprising the ‘pelvic floor dysfunction’ factor in other patient populations may yield useful results.

## Background

The functional gastrointestinal disorders (FGIDs) are biopsychosocial disorders which, like other such disorders for example eating disorders (ED), present difficulties in assessment and measurement [1,2]. Description and categorization of the FGIDs according to the Rome criteria [3] presupposes that clusters of symptoms hold true across different populations; this is despite the fact that the presentation and form of these disorders are affected by a wide range of factors, including physical and psychological comorbidity [4,5].

Factor analysis (confirmatory) seeks to determine if the factors (collections of measured symptoms) confirm what is expected on the basis of pre-established theory and observation. It is perhaps surprising that so few factor analysis studies on the Rome symptom criteria have been conducted. The symptoms of irritable bowel syndrome (IBS) are consistently confirmed in paediatric and adult patients exhibiting functional gastrointestinal symptoms and in community samples [6-10]. The results for functional dyspepsia are less consistent and may involve separate subgroups [10-12].

Despite the high prevalence in ED patients of various gastrointestinal (GI) symptoms consistent with the FGIDs [3], it is not established that these symptoms are truly representative of FGIDs as classified by the accepted standard, namely the Rome criteria. In other words, it is not known whether the GI symptoms commonly found in ED patients can be categorized in the same way as in non-ED patients. This question is clinically relevant, because gastroenterologists and other physicians are frequently referred patients with ED who have gastrointestinal symptoms. If the GI symptoms in this patient group are known to often represent functional GI disorders, as in the general community, and notwithstanding the fact that each patient requires an individual approach, the extent of GI investigation may not need to be as comprehensive as otherwise.

We hypothesized that the specific behaviors, psychopathology and body image concerns characteristic of ED patients would change the clustering or association of GI symptoms, as described by the Rome classification, from that present in non-ED patients and in community samples. The aim of this study was therefore to determine, using factor analysis (FA), whether the GI symptoms that are common in ED patients, hold true to the Rome II FGID classification. Factor analysis was used as it takes into consideration the variability among observed variables. It examines what items correlate together in a multidimensional way and attempts to find an unknown underlying factor that can explain the variability. In other words, FA attempts to find homogeneous clusters or factors amongst a heterogeneous sample.

## Methods

### Patients

185 consecutive eating disorder inpatients admitted to a specialised Unit, specifically for treatment of their eating disorder, in Sydney, Australia, were studied. Eating disorder DSM-IV diagnoses were: anorexia nervosa (N = 84), bulimia nervosa (N = 33) and eating disorder not otherwise specified (EDNOS, N = 68). Comorbidities were low, and included treated diabetes type 1 (N = 2), polycystic ovarian syndrome (2), treated celiac disease (1), and treated bipolar depression (3). All patients otherwise underwent routine clinical evaluation including blood tests (hematology, biochemistry, and thyroid function) and specific investigations to exclude organic gastrointestinal disease where appropriate. All patients gave informed consent. Ethical approval for the study was given by the Northside Clinic Human Ethics Committee.

### Questionnaire

All patients completed the Rome II Modular Questionnaire [5] shortly after admission to hospital. The questionnaire was scored to determine the presence of the Rome II FGID symptom-based diagnoses for the three months prior to admission. Patients did not routinely undergo physiologic testing for a formal diagnosis of those FGIDs requiring such testing, but the symptom criteria were consistent with that particular diagnosis. Patients also completed the Eating and Exercise Examination (EEE) [13]; this included age (years), current and lowest ever BMI kg/m^2^, and eating disorder behaviors, namely objective binge eating, self-induced vomiting, laxative use and excessive exercise. Behaviors were recorded in average days present in the previous 3 months. The definition of objective binge eating was greater than 7 serves of food eaten, associated with feelings that the eating was out of control. The definition of excessive exercise was exercising on greater than 19 days out of 28 days, and was calculated using information on both intensity and duration (kcal/15 mins) [14].

### Data and statistical analysis

The Rome II questionnaire contains 157 items, which would require a very large sample size if all variables were included in the factor analysis. For this reason, measures were taken to reduce the number of variables included in the factor analysis. Specifically, only items showing adequate response variation, defined as symptom presence between 30 and 70% were retained. It is typical to have a ratio of 10 subjects for each item included in a factor analysis [15], and this was the case in the current study after the item pool was reduced. Principal components analysis using varimax rotation was conducted on the responses to the remaining 17 questions (detailed in Table 1). Sampling adequacy was assessed using the Kaiser-Meyer-Olkin (KMO) statistic and the strength of the relationship amongst the variables with Bartlett’s Test of Sphericity.

**Table 1:** Frequency of positive responses to ROME II questions and the factor loadings for the five factors identified using factor analysis*

**Rome II questionnaire**			**Positive**	**Factor 1**	**Factor 2**	**Factor 3**	**Factor 4**	**Factor 5**
**Question number**	**FGID category**	**Question**	**Responses N%**	**Pelvic floor dysfunction**	**Bowel dysfunction**	**Abdominal discomfort**	**Self-induced vomiting**	**Oesophageal discomfort**
38-2	Anorectal	*Unable to empty*	98 53.0	**.765**	.136	.107	.137	.104
24-10	Bowel disorder	*Blocked sensation*	96 51.9	**.753**	.221	.160	.004	.009
38-3	Anorectal	*Difficulty relaxing*	61 33.0	**.720**	-.045	.058	.148	.068
24-7	Bowel disorder	*Incomplete emptying*	112 60.5	**.705**	.110	.177	-.053	-.003
38-1	Anorectal	*Straining*	114 61.6	**.652**	.110	.221	.052	.065
24-11	Bowel disorder	*Need to press*	57 30.8	**.**489	.361	-.242	-.028	-.007
20	Bowel disorder	*Abdominal pain or discomfort*	107 57.8	.091	**.870**	.226	.132	.012
21	Bowel disorder	*Abdominal pain resolves after bowel movement*	83 44.9	.183	**.779**	.350	.049	.224
23	Bowel disorder	*Change in stool form when discomfort starts*	79 42.7	.279	**.744**	.200	.180	.137
12-2	Gastro-duodenal	*Bloating sensation of upper abdomen*	61 33.0	.199	.079	**.848**	-.011	.039
10	Gastro-duodenal	*Pain/discomfort upper abdomen*	95 51.4	.209	.277	**.747**	.030	.235
13	Gastro-duodenal	*Pain stop after bowel movement*	55 29.7	.116	.307	**.711**	-.015	-.038
18	Gastro-duodenal	*Make self vomit*	85 45.9	.071	.093	.000	**.963**	.089
17	Gastro-duodenal	*Vomiting episodes*	89 48.1	.048	.129	-027	**.963**	.098
8	Esophageal	*Heartburn*	65 35.1	-.012	.098	.092	.101	**.872**
6	Esophogeal	*Chest pain*	67 36.2	.064	.069	.028	.042	**.869**
15	Gastro-duodenal	*Burp/belch*	79 42.7	.213	.113	.065	.374	.417

* Factor loadings for the ROME II questions contained in each factor are shown in bold. Only loadings > 0.500 are considered significant loading on a factor.

Factor scores were calculated for each patient for each of the derived factors, and these were correlated against BMI and behavior frequency scores using Spearman correlations. For the behavior scores the average number of days (out of 28) the behavior was present was converted to a 0 to 4 scale (0 = no days, 1 = 1-7 days, 2 = 8-14 days, 3 = 15-21, 4 = 22-28 days). Differences on factor scores between those with or without a specific individual FGID (e.g. IBS, functional bloating or pelvic floor dyssynergia [PFD]) or FGID regional category (e.g. functional oesophageal disorder), or between ED diagnostic groups (anorexia nervosa vs bulimia nervosa vs EDNOS), was explored using ANOVA. Because the specific individual and regional FGID categories, and the factor scores, were all based on Rome II data, alpha was set at a more conservative 0.01 for these latter analyses. All analyses were conducted using SPSS v19 and alpha was set at 0.05 (except for the relationship between FGID and factor scores).

## Results

The clinical details and ED behaviors of the patients are shown in Table 2. The prevalences of regional FGID categories were: functional esophageal disorder 72 (39%), functional gastroduodenal disorder 31 (17%), functional bowel disorder 159 (86%), functional abdominal pain 7 (5%), and functional anorectal disorder 63 (34%). The prevalences of the most common specific FGIDs were: functional heartburn 43 (23%), IBS 77 (42%), functional abdominal bloating 58 (31%), functional constipation 49 (27%), functional proctalgia fugax 37 (20%), and pelvic floor dyssynergia 10 (5%).

**Table 2:** Clinical details and behaviors of 185 eating disorder patients for 3 months prior to admission to hospital

**Clinical details**	**Mean**	**SD**
Age (years)	24.6	8.4
Current BMI (kg/m2)	18.3	3.6
Lowest ever BMI (kg/m2)	15.7	2.7
**Behaviors**	N	%
Restricting food intake daily	183	98.9
Objectively binge eating ( >once/week)	50	27.0
Inducing vomiting (>once/week)	78	42.2
Laxative use (>once/week)	21	16.8
Excessively exercising (> 5 days/week)	39	19.5

Factor analysis of the patients’ Rome II responses revealed five factors explaining 68% of the variance. This was confirmed by Scree plot. The KMO was 0.76 and Bartlett’s test of Sphericity was significant (p < 0.001). The five factors were termed ‘oesophageal discomfort’, ‘self-induced vomiting’, ‘bowel dysfunction’, ‘abdominal discomfort’, and ‘pelvic floor dysfunction’. The factors, the factor loadings and the percentage of patients responding positively to the Rome II questions that contributed to each factor are shown in Table 1.

The percentage of patients responding positively to all questions for each of the factors was: ‘pelvic floor dysfunction’ 22% (5 questions, as ‘need to press’ loading <0.500), ‘bowel dysfunction’ 34% (3 questions), ‘abdominal discomfort’ 20% (3 questions), ‘self-induced vomiting’ 46% (2 questions) and ‘oesophageal discomfort’ 27% (3 questions). For ‘pelvic floor dysfunction’ 56% of patients reported at least 3 of the 5 questions as present.

There were no significant associations of the factor scores with the patient diagnoses, except for the self-induced vomiting factor where bulimia nervosa was significantly different from the anorexia nervosa and EDNOS patients (F(2,182) = 11.550, p < 0.001).

Correlations of the factors with the behaviors and current BMI are shown in Table 3. ‘Oesophageal discomfort’ correlated only with excessive exercise, ‘self-induced vomiting’ correlated with binge eating, laxative use and current BMI, while the other three factors correlated only with laxative use. Relationship with the FGID categories and post hoc ANOVA with IBS, functional abdominal bloating and pelvic floor dyssynergia are shown in Table 4.

**Table 3:** Correlations of factor loadings with current BMI and behaviors present in previous 3 months

**Factors**	**Current BMI**	**Eating disorder behaviors in previous 3 months**			
		**Objective binge eating**	**Self-induced vomiting**	**Laxative use**	**Amount exercise**
Pelvic floor dysfunction				.193**	
Bowel dysfunction				.255**	
Abdominal discomfort				.195**	
Vomiting	.330***	.458***	.800***	.188*	
Oesophageal discomfort					.212**

*p < 0.01, **p < 0.005, ***p < .001.

**Table 4:** Relationship (F values) of factors with specific FGIDs and FGID categories

	**Specific FGIDs**				**FGID categories #**		
**Factors**	**Irritable bowel syndrome**	**Functional abdominal bloating**	**Functional constipation**	**Pelvic floor dyssynergia**	**Oesophageal disorders**	**Bowel disorders**	**Anorectal disorders**
Pelvic floor dysfunction	39.33 ***			13.16***		42.65***	35.46***
Bowel dysfunction	572.78***	44.75***	45.62***			20.17***	
Abdominal discomfort	48.73***	7.29**				7.86*	17.58***
Vomiting	11.15***				8.48**		
Oesophageal discomfort					44.38***		

F values *p < 0.01, **p < 0.005, ***p < 0.001.

# No significant results for FGID categories: gastroduodenal disorders and abdominal pain disorders.

## Discussion

Contrary to our hypothesis, the main finding of this study was that the Rome II classification of specific FGIDs and of FGID regional categories generally holds up to factor analysis of the GI symptoms present in a large sample of ED patients. This was particularly evident for the specific FGIDs of pelvic floor dyssynergia, IBS, functional abdominal bloating and functional constipation, and for three of the FGID regional categories, namely functional oesophageal disorders, functional bowel disorders and functional anorectal disorders. The one exception was the functional gastroduodenal category. Because ED patients exhibit behaviors and psychopathology distinct from non-ED populations, we had hypothesized that the range of GI symptoms prevalent in this group may not conform to the Rome classification of the FGIDs. The clinical relevance of our results is that the GI symptoms in ED patients, once appropriate investigations have been performed as indicated, are very likely to represent the same FGIDs seen in non-ED patients.

The factor we termed ‘oesophageal discomfort’ emerged as a separate factor, and consisted of heartburn and chest pain of presumed oesophageal origin. This factor was associated with excessive exercise only and this is entirely consistent with reports of heartburn and chest pain, and disorders such as gastroesophageal reflux and altered oesophageal motility, occurring in runners [16]. The factor ‘self-induced vomiting’ was to be expected in this sample of ED patients, and not surprisingly was more common in patients with bulimia nervosa, as this phenomenon is included in the diagnostic criteria for ED. In the Rome II questionnaire, if vomiting is self-induced it is discounted for the diagnosis of functional vomiting. This factor, not part of the Rome classification, was associated with vomiting questions only and not with other oesophageal symptoms.

The two separate factors we termed ‘bowel dysfunction’ and ‘abdominal discomfort’ were both significantly associated with the FGID category of functional bowel disorder. The factor termed ‘bowel dysfunction’ related to a change in stool pattern rather than bloating and appeared to be consistent with IBS. This is confirmed by the highly significant relationship between ‘bowel dysfunction’ and IBS (Table 4). The factor ‘abdominal discomfort’, although also associated with IBS, was specifically associated with the FGID category of anorectal disorder. This finding is consistent with our recent report of a relationship between prolonged rectal balloon expulsion time and abdominal distension in patients with constipation [17]

The factor we termed ‘pelvic floor dysfunction’ is consistent with the FGID category of functional anorectal disorders and with the specific FGID of pelvic floor dyssynergia. In the Rome II criteria, a diagnosis of pelvic floor dyssynergia requires a concomitant diagnosis of functional constipation, while a diagnosis of functional constipation cannot be made if IBS is present. It can be therefore difficult to obtain a diagnosis of pelvic floor dyssynergia, especially when IBS is so common among ED patients. In this study, however, factor analysis has revealed 5 questions usually associated with pelvic floor dyssynergia and functional constipation to cluster together independent of the presence or absence of IBS. An alternative term for the pelvic floor dysfunction factor could have been functional obstructed defecation or *anismus*, as we have no information about urinary or vaginal pelvic floor problems in these patients. Anismus is a form of functional obstructed defecation and can cause constipation [18] and painful defecation. It is more common in women than in men, and sometimes is associated with sexual abuse [19].

Finally, the correlation of laxative abuse with most of the factors is likely to reflect the higher prevalence of laxative use and abuse among ED patients before admission to hospital for treatment of their eating disorder. However there is little information about laxative use in other FGID populations.

## Conclusions

Factor analysis of gastrointestinal symptoms in ED patients is generally consistent with the Rome II classification of FGIDs. Our findings, in a different patient group, therefore support the validity of this symptom-based classification. These results are also clinically relevant, in that they provide the strongest evidence to date that the GI symptoms common in patients with ED indeed reflect the presence of FGIDs. However, pelvic floor symptoms in the absence of functional constipation and separate from IBS, are a prominent feature of ED patients. These symptoms therefore warrant further research, and should be evaluated in different populations, including women in the community.

## Competing interests

The authors report no competing interests, financial or otherwise.

## Authors’ contributions

SA is responsible for, data analysis, content and writing of the paper. JK is responsible for the content and writing of the paper. Both authors read and approved the final manuscript.

## Pre-publication history

The pre-publication history for this paper can be accessed here:

http://www.biomedcentral.com/1471-230X/13/38/prepub
